# Viral protein engagement of GBF1 induces host cell vulnerability through synthetic lethality

**DOI:** 10.1083/jcb.202011050

**Published:** 2022-10-28

**Authors:** Arti T. Navare, Fred D. Mast, Jean Paul Olivier, Thierry Bertomeu, Maxwell L. Neal, Lindsay N. Carpp, Alexis Kaushansky, Jasmin Coulombe-Huntington, Mike Tyers, John D. Aitchison

**Affiliations:** 1 Center for Global Infectious Disease Research, Seattle Children’s Research Institute, Seattle, WA; 2 Institute for Research in Immunology and Cancer, Université de Montréal, Montreal, Quebec, Canada; 3 Center for Infectious Disease Research, Seattle, WA; 4 Department of Pediatrics, University of Washington, Seattle, WA; 5 Department of Biochemistry, University of Washington, Seattle, WA

## Abstract

Viruses co-opt host proteins to carry out their lifecycle. Repurposed host proteins may thus become functionally compromised; a situation analogous to a loss-of-function mutation. We term such host proteins as viral-induced hypomorphs. Cells bearing cancer driver loss-of-function mutations have successfully been targeted with drugs perturbing proteins encoded by the synthetic lethal (SL) partners of cancer-specific mutations. Similarly, SL interactions of viral-induced hypomorphs can potentially be targeted as host-based antiviral therapeutics. Here, we use GBF1, which supports the infection of many RNA viruses, as a proof-of-concept. GBF1 becomes a hypomorph upon interaction with the poliovirus protein 3A. Screening for SL partners of *GBF1* revealed *ARF1* as the top hit, disruption of which selectively killed cells that synthesize 3A alone or in the context of a poliovirus replicon. Thus, viral protein interactions can induce hypomorphs that render host cells selectively vulnerable to perturbations that leave uninfected cells otherwise unscathed. Exploiting viral-induced vulnerabilities could lead to broad-spectrum antivirals for many viruses, including SARS-CoV-2.

## Teaser

Using a viral-induced hypomorph of GBF1, Navare et al. demonstrate that the principle of synthetic lethality is a mechanism to selectively kill virus-infected cells.

## Introduction

The principle of synthetic lethality offers an opportunity for selectively targeting virus-infected cells by drugging synthetic lethal (SL) interactors of virus-targeted multifunctional protein hubs (Mast et al., 2020). Synthetic lethality occurs between two genes when a loss-of-function mutation in either gene has little impact on cell viability, but becomes detrimental when paired together resulting in cell death (Dobzhansky, 1946; Hartwell et al., 1997). Such lethal genetic combinations, known as “SL pairs” (Nijman, 2011), are one of many forms of genetic interactions that can occur within cells (Boone et al., 2007; Dixon et al., 2009; Drees et al., 2005; Horlbeck et al., 2018). The existence of synthetic lethality reveals important aspects of the genetic architecture of cells, demonstrating the presence of genetic buffering in organisms due to functional redundancy (Horlbeck et al., 2018; McManus et al., 2009). Synthetic lethality–inspired anticancer therapy provides avenues for improved drug specificity and efficacy at lower doses, thereby limiting side effects (Beijersbergen et al., 2017). Here, we extend the application of this synthetic lethality principle to host-derived antiviral targets.

Virus infection perturbs host protein–protein interactions (PPIs), usurping normal protein functions and rewiring normal host PPI networks. Host proteins are considered proviral if loss-of-function renders the host cell resistant to infection and antiviral if loss-of-function improves cell permissibility to infection (Mast et al., 2020). Infected cells exhibit altered metabolic requirements (Thaker et al., 2019), signaling pathways (Gaur et al., 2011), and intracellular transport pathways (Belov et al., 2007), as well as other morphological and molecular characteristics (Mirabelli et al., 2021) relative to the uninfected cells. In such situations, infected cells may depend on a different complement of proteins than their uninfected counterparts (Mast et al., 2020). This state-specific vulnerability may be a target for host-based therapeutics based on the well-established principle of synthetic lethality. For example, if two host cell proteins have an SL relationship and the function of one protein is hijacked by a viral protein, then cells may become dependent on the function of the second protein. In contrast, cells that are not altered by the virus, i.e., those that are uninfected, will be unimpacted by disrupting the second protein, since the elimination of a single half of the SL pair does not result in a phenotype. Rational targeting of SL protein pairs in which the function of one partner is reduced specifically in the infected cell, a situation analogous to a mutant gene in cancer, is a novel framework for taking advantage of the intrinsic differences of infected cells to achieve selective targeting.

We hypothesize that viral-host PPIs generate protein-based, viral-induced (vi)-hypomorphs of host factors in infected cells, thereby specifically sensitizing infected cells to targeting genetically interacting (SL/synthetic sick) partners of these vi-hypomorphs. To test this hypothesis, we selected the Golgi-specific brefeldin A-resistance guanine nucleotide exchange factor (GBF1; Claude et al., 1999) as a potential prototypical vi-hypomorph because it is a critical proviral host factor for the replication of several families of RNA viruses, including *Picornaviridae, Coronaviridae, Flaviviridae*, *Herpesviridae*, *Filoviridae*, and *Rioviridae* (Belov et al., 2008; Carpp et al., 2014; Farhat et al., 2018; Goueslain et al., 2010; Lanke et al., 2009; Martínez et al., 2019; Verheije et al., 2008; Yamayoshi et al., 2010).

Many RNA viruses encode proteins that bind GBF1 directly, including the nonstructural proteins 3A of poliovirus (Belov et al., 2008; Teterina et al., 2011) and coxsackievirus (Wessels et al., 2006a; Wessels et al., 2006b), and nonstructural protein 5 of dengue virus (Carpp et al., 2014). Recently, two SARS-CoV-2 proteins, membrane (M) and orf6, were also identified to directly bind or reside in close proximity to GBF1, respectively (Laurent et al., 2020 *Preprint*; Stukalov et al., 2021). In the case of poliovirus infection, 3A redistributes GBF1 to viral replication complexes during early stages of replication and subverts its guanine nucleotide exchange factor (GEF) function in infected cells (Belov et al., 2007, 2008; Richards et al., 2014), suggesting that poliovirus protein 3A may attenuate GBF1’s normal function creating a vi-hypomorph, rendering cells susceptible to disruption of proteins synthetically lethal with *GBF1*. Here, we provide proof of concept that SL partners of vi-hypomorphs can be targeted to selectively eliminate infected cells while sparing uninfected cells. We do this by performing a genome-wide chemogenomic CRISPR screen to identify SL partners of *GBF1*, validating the top candidates, and demonstrating that shRNA-mediated silencing of the *GBF1* SL interacting partner, *ARF1*, selectively kills cells expressing poliovirus protein 3A.

## Results and discussion

**Figure 1.**
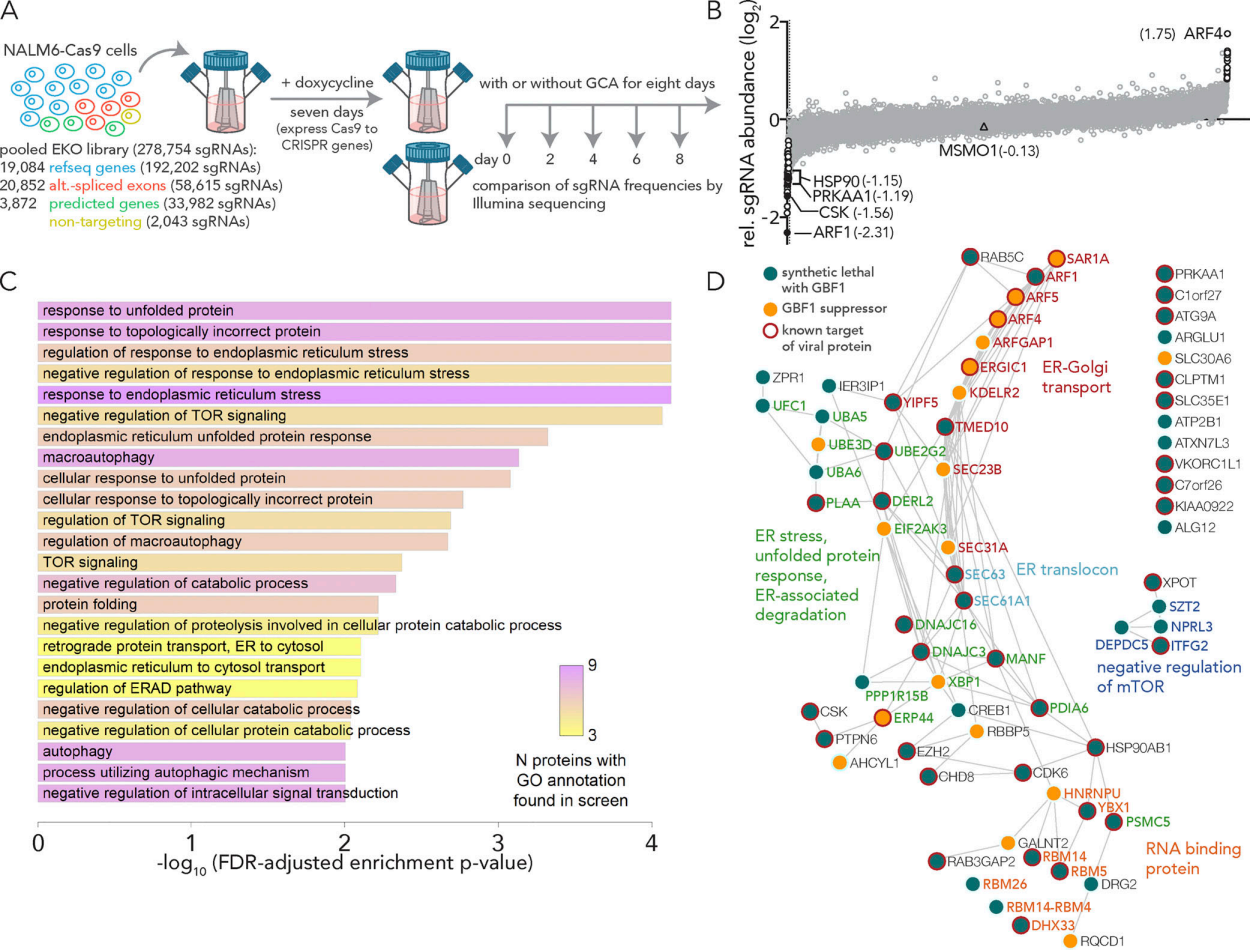
**A chemogenomic screen identifies synthetic lethal partners of GBF1. (A)** A schematic of the experimental design for chemogenomic screening with the GBF1 inhibitor golgicide A (GCA). A CRISPR extended knockout (EKO) library of NALM6-Cas9 cells was treated with 2 µg/ml doxycycline to induce individual gene knockouts via Cas9 expression. The pooled library was split into individual flasks and grown over an 8-d period in the presence or absence of 4 µM golgicide A (GCA). Following incubation, sgRNA frequencies were measured using Illumina sequencing, and log_2_ fold changes between GCA and control samples were compared. **(B)** A plot of relative sgRNA frequencies of all genes with genes passing a 0.05 FDR cutoff in white and black filled circles. The 53 genes with negative sgRNA fold change from GCA treatment represent putative SL interactors of *GBF1*. The 17 genes with overrepresented sgRNAs and positive sgRNA fold change represent putative *GBF1* suppressors that may confer protection against GCA. Triangle represents no change in abundance and denotes MSMO1. **(C)** Gene ontology functional enrichment analysis of SL partners of *GBF1*. The 53 putative SL partners of *GBF1* were analyzed in clusterProfiler against the entire KO gene collection from the CRISPR library to functionally classify the SL genes. Significantly enriched gene ontologies are plotted and ranked by their -log_10_, FDR-adjusted enrichment P value. The number of putative SL genes in each gene ontology is coded by the heatmap and ranges from 3 (yellow) to 9 (pink). **(D)** A combined PPI network of the 53 synthetic lethal interactors of *GBF1* (green circles) and the 17 *GBF1* suppressors (orange circles) was obtained from the STRING database and visualized using Cytoscape. Edges between two circles denote evidence-based interaction between the connecting proteins. Circles with red outlines highlight known targets of viral proteins, as per the VirHostNet (v2.0) virus-host PPIs database. Gene names of the proteins and their gene ontology functions are color matched.

To identify putative SL partners of *GBF1*, we screened a high-complexity extended-knockout CRISPR library of 278K single guide RNAs (sgRNAs) that target 19,084 RefSeq genes, 20,852 alternatively spliced genes, and 3,872 predicted genes, among additional controls, in NALM-6 human B-cell precursor leukemia cells (Fig. 1 A; Bertomeu et al., 2018). These cells harbor a genomic doxycycline-inducible Cas9 that enables regulatable, uniform, and robust gene silencing across the pooled library (Wang et al., 2014). Relative changes in sgRNA frequencies were obtained from sequencing populations of the CRISPR libraries cultured in the presence or absence of Golgicide A (GCA), a potent and specific inhibitor of the GEF activity of GBF1 (Fig. 1 A; Sáenz et al., 2009). sgRNA frequencies were determined by sequencing, and relative fold changes in sgRNA abundances between GCA- and mock-treated samples are reported (Table S1 and Fig. 1 B).

Our CRISPR screen identified 53 underrepresented genes and 17 overrepresented genes in the GCA-treated samples relative to controls (Fig. 1 B; white and black filled circles; FDR = 0.05). Underrepresented genes reflect cells depleted from the population and define putative SL partners of GBF1. Putative SL interactors of *GBF1* are likely functionally redundant with *GBF1*, an attribute of the genetic interactions between SLs that offers buffering in the event of a loss of function of one of the SL genes (Hartman et al., 2001; Mast et al., 2020). Overrepresented genes reflect cells enriched in the population. These overrepresented genes may counter the harmful effects of *GBF1* inhibition and are termed “*GBF1* suppressors” (Van Leeuwen et al., 2016). Functional enrichment analysis of the 53 putative SLs of GBF1 identified 29 genes involved in the misfolded protein-triggered ER stress response and 19 genes in the early secretory pathway, reflecting well-characterized GBF1 biology (Fig. 1, C and D; Beller et al., 2008; Citterio et al., 2008; Donaldson and Jackson, 2011; Guo et al., 2008; Manolea et al., 2008; Sáenz et al., 2009; Soni et al., 2009). As evident by the PPI network, the 53 *GBF1*-SL partners and the 17 *GBF1* suppressors are functionally related and can be grouped into a few distinct functional clusters (Fig. 1 D). For example, one cluster of *GBF1*-SL pairs is enriched in ER stress, unfolded protein response, and ER-associated protein degradation pathways, while 8 out of the 17 *GBF1* suppressors contribute to ER-Golgi vesicular transport (Fig. 1 D). *GBF1*-SL pairs also include a cluster of RNA-binding proteins, and members of the KICSTOR (Wolfson et al., 2017) and DEPTOR (Peterson et al., 2009) complexes that negatively regulate mTOR signaling (Fig. 1 D). Several genes from both lists possess GTPase activity, e.g., the *GBF1*-SL partners: *ARF1*, *TMED10*, *DRG2*, *RAB5C*, *YIP5*, *RAB3GAP2*, and the *GBF1* suppressors: *ARF1GAP1*, *SAR1A1*, *ARF4*, and *ARF5* (Fig. 1 D). Just over half of the *GBF1*-SL partners and suppressors identified in our screen are directly targeted by viral proteins (Fig. 1 D; circles with red boundaries).

The top SL candidate ADP-ribosylation factor 1 (*ARF1*), a small GTPase that regulates the recruitment and assembly of COP I on Golgi and ERGIC membranes (Liang and Kornfeld, 1997), is activated by GBF1 which facilitates GDP to GTP exchange on ARF1. GBF1-activated ARF1 has broad cellular activities, including recruitment of coat proteins and lipid-modifying enzymes to facilitate secretory cargo transport (Claude et al., 1999; Donaldson and Jackson, 2011; Kawamoto et al., 2002). Consistent with our observations, a negative genetic interaction exists between *ARF1* and the yeast GBF1-ortholog, guanine nucleotide exchange on ARF1 (*GEA1*; Surma et al., 2013), and *GEA1* overexpression rescues an *arf1*∆ growth defect (Chantalat et al., 2003).

ARF4, a class II ARF implicated in endosomal morphology and retrograde transport to the Golgi (Nakai et al., 2013), was the top overrepresented gene (Fig. 1 B). Consistent with our observation of a protective role for ARF4 against GCA toxicity, ARF4 was found to be protective against the Golgi disrupting agent Brefeldin A (BFA; Reiling et al., 2013) which targets the ARF GEFs GBF1, BIG1 (Morinaga et al., 1997; Morinaga et al., 1996), and BIG2 (Togawa et al., 1999). ARF4 depletion stimulates poliovirus replicon replication and suppresses deleterious effects on the poliovirus replicon by BFA-mediated GBF1 inhibition (Moghimi et al., 2020). ARF4 knockdown (KD) protects against infection by other human pathogens including *Chlamydia trachomatis* and *Shigella flexneri* (Reiling et al., 2013), consistent with an important role for ARFGEFs, such as GBF1, for these intracellular pathogens.

**Figure 2.**
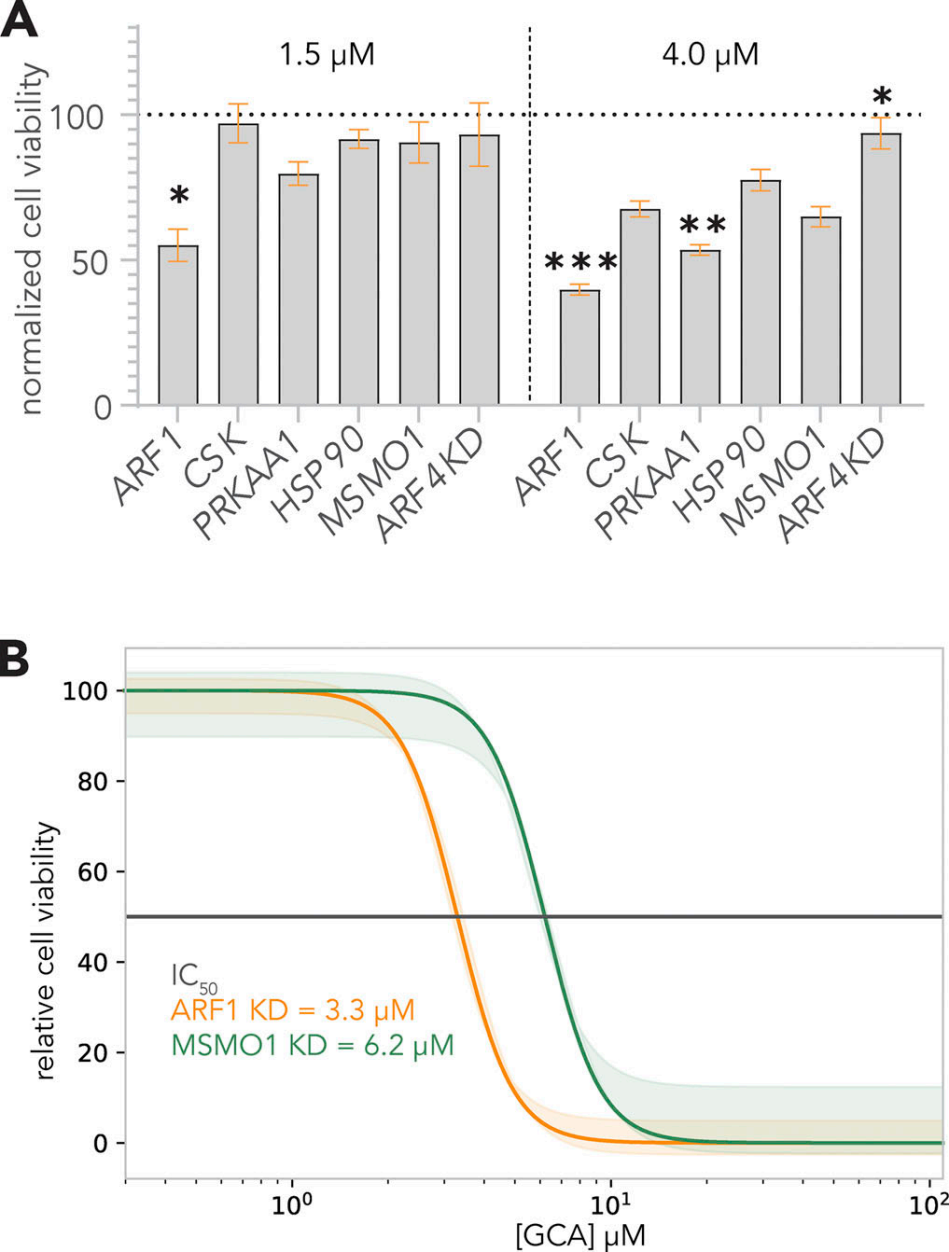
**Validation of putative synthetic lethal interactions in HeLa cells. (A)** *ARF1* displays a robust synthetic lethal interaction with *GBF1*. *ARF1*, *HSP90*, *CSK*, *PRKAA1*, the control gene *MSMO1*, and the top *GBF1* suppressor gene *ARF4* were silenced in HeLa cells with shRNA-mediated lentivirus transductions and incubated with golgicide A (GCA) at a concentration of 1.5 µM (left panel) or 4 µM (right panel) or DMSO alone for 48 h. CellTiter Blue reagent was added and fluorescence measurements were collected. The percent viability at each GCA concentration was calculated by dividing the fluorescence from a GCA-treated sample by its matched DMSO alone-treated control to compensate for DMSO solvent effects. Changes in cell viabilities for each knockdown (KD) cell line were then determined by comparing the respective percent viabilities to the *MSMO1* KD control using a Brown Forsythe and Welch ANOVA multiple comparison test (Brown and Forsythe, 1974; Welch, 1951), with statistically significant differences indicated as: * if P value < 0.01; ** if P value < 0.001; *** if P value < 0.0001. Error bars represent the SEM from three biological replicates. **(B)** *ARF1* KD cells show enhanced sensitivity in a GCA dose-response curve. A 200 µM GCA working solution in DMSO was serially diluted and co-plated with 20,000 cells per well of *ARF1* KD and *MSMO1* KD cells in a 96-well plate, with final GCA concentrations ranging from 0–100 µM. After 48 h, cell viability was measured with CellTiter Blue and the normalized fluorescence, relative to DMSO-treated samples, was calculated using the smallest and largest mean values to define 0 and 100%, respectively. A dose response curve of the normalized fluorescence was plotted against GCA concentration and IC_50_ values were calculated. The dose response curve and its 90% confidence interval were plotted from the results of four biological replicates per treatment.

To validate putative GBF1-SL pairs in HeLa cells, we performed KDs in the presence of GCA. We prioritized potential druggable candidates (black filled circles; Fig. 1 B) using the Drug Gene Interaction Database (http://dgidb.org/search_categories; Cotto et al., 2018). In addition to *ARF1*, we selected: heat-shock protein 90 (*HSP90*), a protein chaperone with ATPase activity (Rowlands et al., 2010); C-terminal Src kinase (*CSK*) which negatively regulates Src family kinases and has roles in cell growth, differentiation, migration and immune responses (Okada, 2012); and protein kinase, AMP-activated, alpha 1 (*PRKAA1*), the catalytic subunit of the 5′-prime-AMP-activated protein kinase (AMPK) with roles in regulating cell stress and metabolism (Fig. 2 A; Sanli et al., 2014). We also included methylsterol monooxygenase 1 (*MSMO1*) as a control because it did not show depletion or enrichment in sgRNA abundance (triangle; Fig. 1 B), and the top overrepresented GBF1 suppressor, *ARF4* (Fig. 1 A and Table S1). SL effects of combining GCA with shRNA-mediated depletion were observed in *ARF1* KD cells with only 40% (at 4 µM) or 50% (at 1.5 µM) viability as compared to the DMSO alone–treated cells (Fig. 2 A). When viability (normalized to the matched DMSO alone–treated cells) of each KD cell line was compared to that of the *MSMO1* KD control, the decrease in viability was statistically significant for *ARF1* KD at both concentrations (ANOVA P value <0.01 and 0.0001 at 1.5 and 4 µM, respectively), consistent with the results of the chemogenomic screen (Fig. 1). A GCA dose-response assay monitoring cell growth inhibition as a function of GCA concentration showed a nearly twofold reduction in IC_50_ value for *ARF1* KD cells as compared to the control (Fig. 2 B), further validating the SL interaction between *GBF1* and *ARF1*.

**Figure 3.**
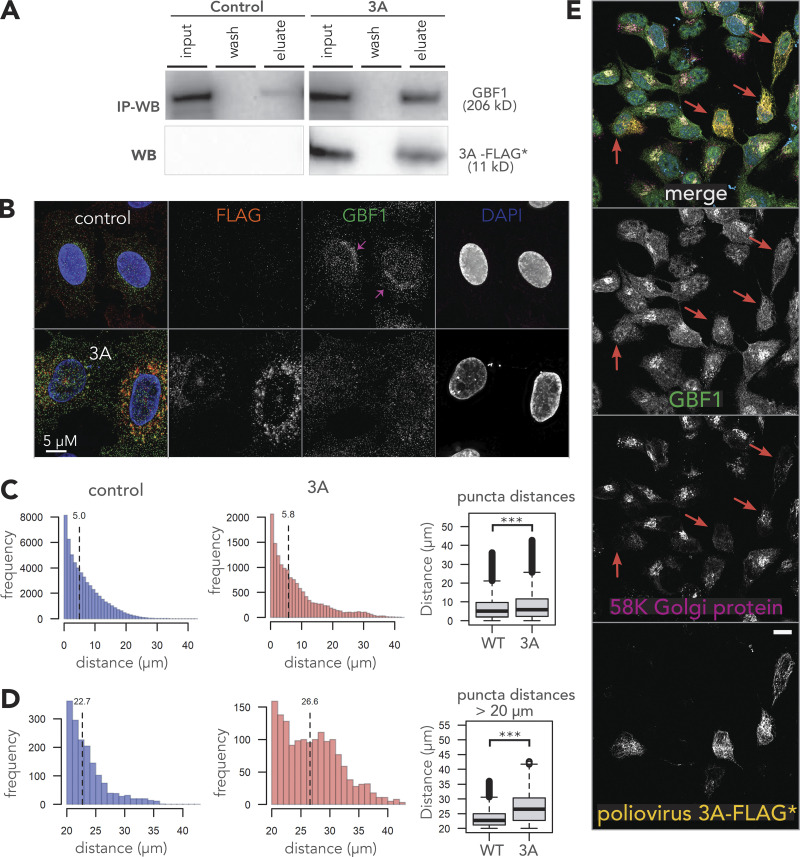
**Poliovirus nonstructural protein 3A induces a *vi-hypomorph* of GBF1. (A)** Poliovirus 3A physically interacts with GBF1. HeLa cells were transfected with FLAG* tagged poliovirus 3A or an empty control plasmid for 24 h. Equal amounts of lysates were prepared, and immunoaffinity enriched proteins bound to 3A-FLAG* protein. Affinity captured proteins were eluted and resolved on SDS-PAGE along with 1% of the total input lysate and the final wash. Resolved proteins were transferred to a PVDF membrane and immunoblotted using anti-GBF1 (top panel) and anti-FLAG (bottom panel) antibodies. This experiment was performed in triplicate. **(B)** Poliovirus 3A disperses GBF1 away from its perinuclear localization. HeLa cells transfected with FLAG* tagged poliovirus 3A, or an empty control plasmid were fixed, stained with fluorescently labeled antibodies against FLAG and GBF1, and imaged by wide-field fluorescence microscopy. **(C and D)** Images of GBF1 were analyzed and the distances of each GBF1 punctum to the nearest nucleus was determined and plotted across all distances (C) and those between 20 and 40 µM (D) for 42 control cells and 13 cells transfected with 3A-FLAG*. The corresponding box plots show statistically significant differences in GBF1 distribution between the two samples with *** representing a P value < 0.0001. **(E)** Poliovirus 3A induces fragmentation of the Golgi. HeLa cells were transfected with FLAG* tagged poliovirus 3A, and after 24 h fixed, stained with fluorescently labeled antibodies against FLAG (Red), GBF1 (green) and a Golgi marker protein 58 k (magenta). The nucleus was visualized by DAPI (cyan). Poliovirus 3A-FLAG* expressing cells are highlighted with arrows. Bar, 10 µm. Source data are available for this figure: SourceData F3.

The premise of SL-specific antivirals is that a viral infection disrupts normal protein functions consequently generating vi-hypomorphs in infected cells. As a result, the infected cells may become selectively vulnerable to perturbations that target SL partners of the vi-hypomorphs. We tested this hypothesis in the context of cells expressing poliovirus 3A. Poliovirus 3A interacts with GBF1 and recruits it to sites of replication during poliovirus infection (Belov et al., 2007; Belov et al., 2008; Richards et al., 2014). We expressed 3A alone to avoid the confounding effects of the multitude of changes induced by viral infection, and to test the formation of a GBF1 vi-hypomorph in a simpler yet relevant model system. The dynamics of GBF1-3A interactions observed during viral infection, including GBF1-mediated ARF1 activation and translocation, are retained in cells ectopically expressing the viral protein alone (Belov et al., 2005; Belov et al., 2007; Richards et al., 2014; Wessels et al., 2006a). N-terminal, FLAG*-tagged 3A (Teterina et al., 2011), and associated proteins were affinity purified from HeLa cells and analyzed by Western blotting (Fig. 3 A). A band of ∼10 kDa was detected in the eluate corresponding to the 3A-FLAG* protein and a slower migrating, high molecular weight band of ∼200 kDa, corresponding to GBF1, was detected in the eluate of GBF1 affinity purified from cells expressing 3A-FLAG*, but not from control cells (Fig. 3 A). This observation confirmed that the ectopically produced 3A-FLAG* protein retained its ability to physically interact with GBF1, as reported previously (Teterina et al., 2011).

We tested if the physical interaction between 3A and GBF1 had consequences for GBF1 function or localization, which would suggest the generation of a GBF1 hypomorph. HeLa cells expressing 3A-FLAG* were fixed and immunostained with α-FLAG-647 (red) and α-GBF1-488 (green), antibodies (Fig. 3 B). In control cells, GBF1 was visualized as puncta enriched in a juxtanuclear position consistent with its Golgi localization (Fig. 3 B; red arrows), whereas GBF1 puncta were redistributed throughout the cytoplasm in 3A expressing cells (Fig. 3, B–D). Coxsackievirus 3A, a closely related homolog of poliovirus 3A, also induces similar changes in GBF1 localization (Wessels et al., 2006b). This re-distribution of GBF1 coincided with Golgi fragmentation as detected by the Golgi membrane marker 58K Golgi protein and as previously observed for disruption of GBF1 function (Fig. 3 E; Chan et al., 2019; Citterio et al., 2008; Doedens and Kirkegaard, 1995; Sáenz et al., 2009). We interpret these results to reflect the formation of a 3A-induced GBF1 hypomorph and consequential disruption of the Golgi apparatus.

**Figure 4.**
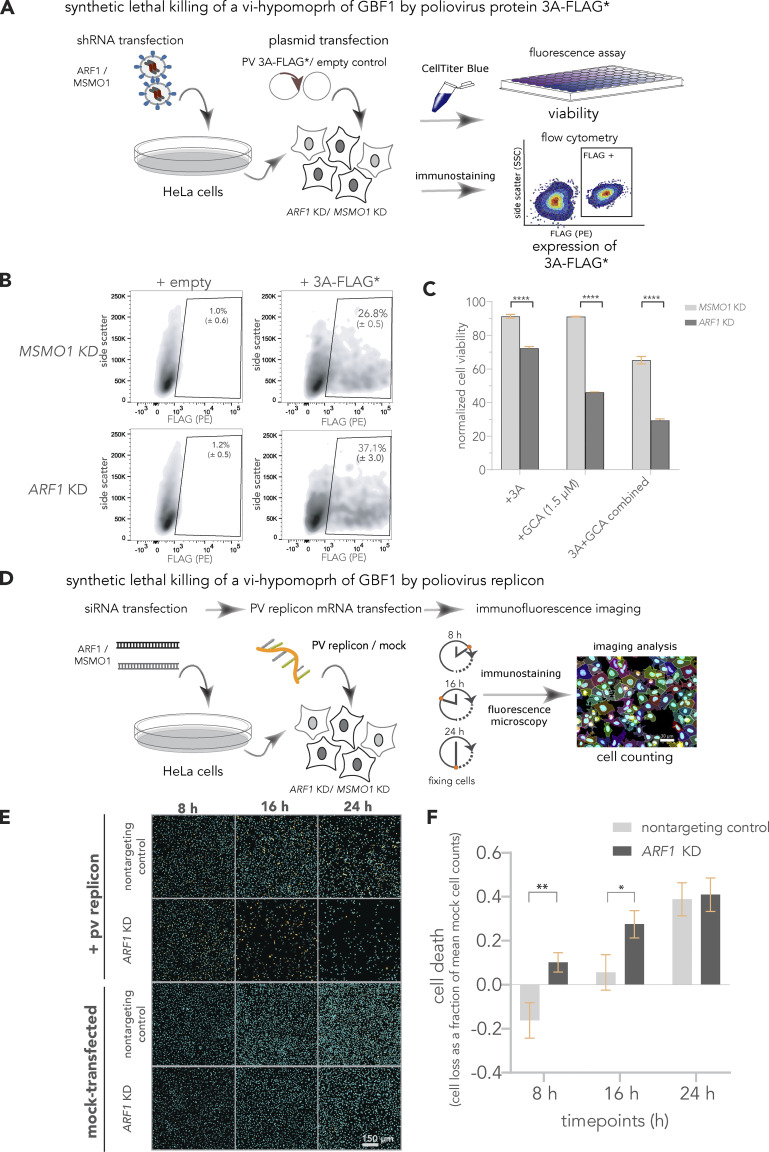
**The 3A induced hypomorph of GBF1 sensitizes cells to *ARF1*-*GBF1 synthetic lethality.* (A–C)** Synthetic lethal killing of a vi-hypomorph of GBF1 by poliovirus protein 3A. **(A)** *ARF1* and *MSMO1* were stably silenced in HeLa cells and transfected with FLAG*-tagged poliovirus 3A. Cell viabilities of *ARF1* KD and *MSMO1* KD cells transfected with 3A-FLAG* or an empty plasmid control were measured with CellTiterBlue. **(B)** The population (Side scatter [y-axis]) positively stained with R-phycoerythrin (PE)-conjugated α-FLAG (x-axis) was quantified to determine the number of cells positive for 3A-FLAG* in *ARF1* KD and *MSMO1* KD cells by flow cytometry, post 24 h. **(C)** Poliovirus 3A induces cell death in *ARF1* KD cells. The viabilities of *ARF1* KD and *MSMO1* KD cells treated with 3A-alone or GCA-alone (1.5 μM) and a combination of 3A and GCA were measured using CellTiter Blue and plotted as a percentage of total cells. The percentage of viable cells was calculated by dividing the absolute fluorescence values of treated samples by the matched controls (un-transfected, DMSO-alone treated KD cells). A multiple unpaired *t* test was used to compare percent viability between the 3A-treated *ARF1* KD and *MSMO1* KD cells with **** representing P value < 0.000001. Error bars represent the SEM from six biological replicates. **(D–F)** Synthetic lethal killing of a vi-hypomorph of GBF1 by poliovirus replicon. **(D)** HeLa cells were transiently transfected with siRNAs targeting *ARF1* or a nontargeting control. The KD or control cells were transfected with 25 ng of poliovirus replicon mRNA, and cells were incubated for 24 h. Cells were harvested and fixed at 8, 16, and 24 h post-transfection, immunostained, and imaged by fluorescence microscopy. **(E)** Representative immunofluorescence images of nontargeting and *ARF1* KD cells transfected with poliovirus replicon or mock at 8, 16 and 24 h post-transfection. DAPI (cyan) was used to stain cell nuclei and the viral 3A-FLAG* protein was immunostained with a primary antibody against the FLAG tag (orange). **(F)** Quantification of cell depletion. DAPI signal was used to count the total number of imaged cells. Cell counts of the replicon-transfected samples were subtracted from and divided by the average cell counts of time-matched mocks to quantify cell death (cell loss as a fraction of mean mock cell counts) post-transfection over the course shown. A multiple *t* test was used to compare cell death between poliovirus replicon-transfected *ARF1* KD and nontargeting controls at each time point. Statistically significant differences were observed at 8 h (P value <0.05 indicated as *) and at 16 h (P value <0.01, indicated as **). Error bars represent the SEM from nine biological replicates.

We asked if the 3A-induced hypomorph of GBF1 was sufficient to sensitize cells to ARF1 depletion and expose the SL interaction between *GBF1* and *ARF1*. HeLa cells were first treated with shRNA to *ARF1*, or, as a control, shRNA to *MSMO1* (Fig. 4 A). The depletion of ARF1 was evaluated by Western blotting (Fig. S2), 3A-FLAG* was detected by flow cytometry, and cell viability was measured using the CellTiter Blue assay as before (Fig. 4, A and B). The viability of the *ARF1* KD cells was significantly decreased by 3A-FLAG* expression as compared to controls (*MSMO1* KD; Fig. 4 C). Importantly, this decrease in cell viability, 30% ± 3.5, (Fig. 4 C) was comparable to the percent of *ARF1* KD cells expressing 3A-FLAG*, 37% ± 3.0 (Fig. 4 B). This supports our hypothesis that 3A expression induces a GBF1 hypomorph, exposing the *GBF1*-*ARF1* SL relationship, which allowed selective killing of 3A expressing cells by KD of *ARF1* (Fig. 4, B and C). Consistent with this hypothesis, combining 3A expression with sublethal amounts of GCA further exacerbated the *GBF1*-*ARF1* SL effect (Fig. 4 C).

We asked if the 3A-induced hypomorph of GBF1 was sufficient to sensitize cells to SL killing in the context of a viral infection. For these experiments, we used a poliovirus replicon encoding 3A-FLAG* and other poliovirus genes, except with gene encoding *Renilla* luciferase in the place of capsid (Belov et al., 2007; Teterina et al., 2011). Cells were treated with siRNA against *ARF1* or a nontargeting control and KD was evaluated by Western blotting (Fig. S2). The poliovirus replicon was transfected into *ARF1* KD and nontargeting control cells, and the number of cells counted after 8, 16, or 24 h (Fig. 4 D). As replication of the poliovirus replicon progressed, *ARF1* KD cells were selectively depleted at 8 and 16 h compared to the nontargeting control (Fig. 4, E and F). However, by 24 h cell depletion in the nontargeting control was comparable to *ARF1* KD (Fig. 4 E), consistent with previously reported cytotoxicity of this poliovirus construct on cells (Belov et al., 2008; Suk Oh et al., 2009). A recent study by Moghimi et al. (2020) revealed that replication of the poliovirus replicon was also significantly reduced in *ARF1* KD cells when treated with BFA. In the light of our study, we interpret their results to reflect the impact of both GBF1 inhibition by BFA and the formation of a GBF1 hypomorph, exposing the *GBF1*-*ARF1* synthetic lethality. Our results demonstrate that SL relationships can be exploited to kill virus-infected cells early in infection and well before virus-mediated cell death.

**Figure 5.**
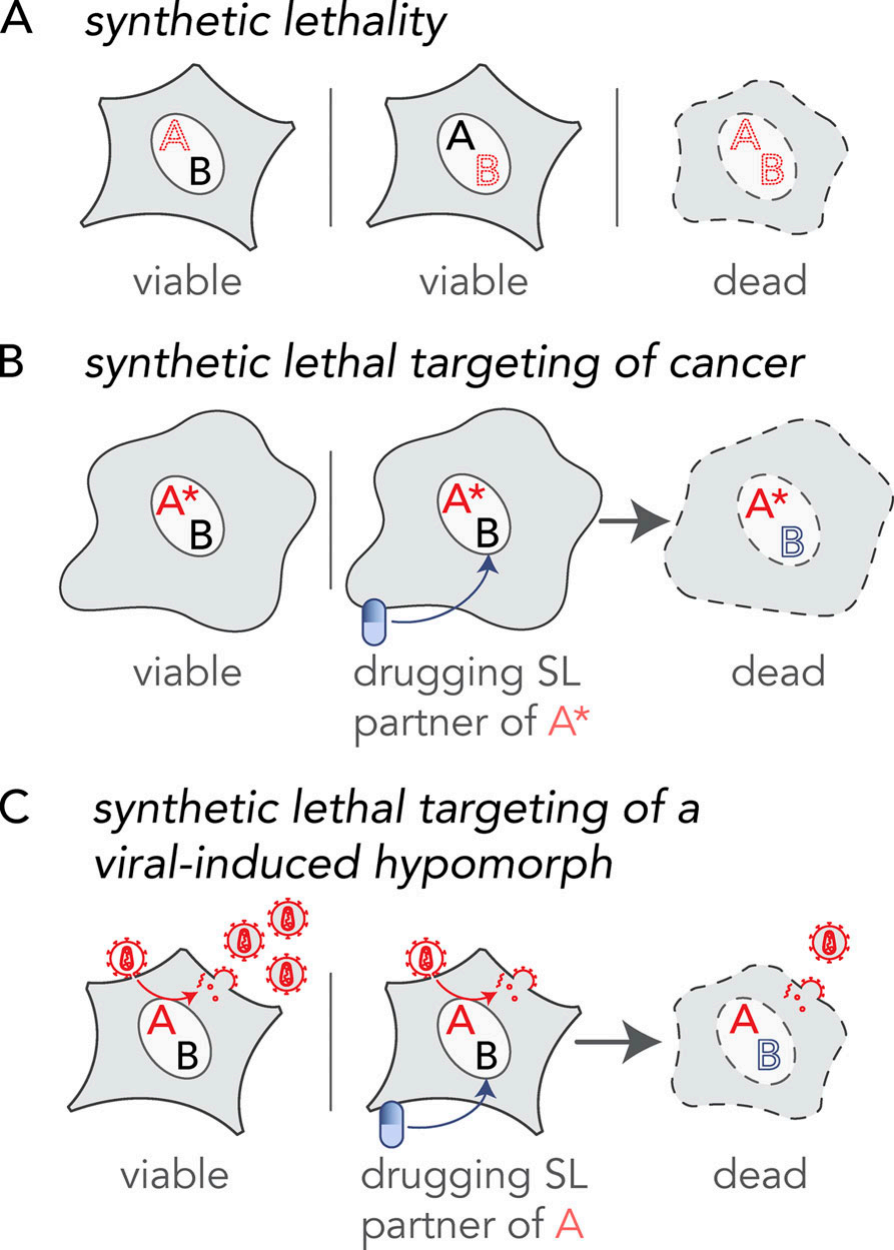
**Extending the principle of synthetic lethal interactions to a virus-induced hypomorph. (A)** Synthetic lethality is an extreme negative genetic interaction occurring between two genes. Here, genes “A” and “B” are not essential, and the cell remains viable upon the loss of either gene, depicted by red dotted outline of “A” or “B”, individually. However, when these deletions are combined in a single cell, as visualized in the third panel, this double loss of function critically impairs the cell, resulting in its death. Such gene-gene combinations are termed synthetic lethal (SL) partners. **(B)** The principle of synthetic lethality has been successfully exploited in the development of certain cancer therapies by targeting the synthetic lethal partner of the cancer-causing oncogene, depicted by a red “A.” In the cancerous cell, gene “A” has been mutated, depicted as “A*,” leading to an enhanced dependency by the cancer cell for its synthetic lethal partner “B.” Drugs that target the otherwise nonessential gene B induce cell death when combined with its SL partner, A*. Therefore, inhibiting the function of B can selectively kill cancerous cells while sparing noncancerous bystander cells. **(C)** Like the example in cancer, a viral infection provides opportunities for specifically targeting infected cells by synthetic lethality. When a cell is infected, host factors, depicted as the red letter “A,” are recruited by viral proteins to support viral reproduction. The normal function of the host factor is thus attenuated by the presence of the virus, inducing a hypomorph, red letter “A,” which sensitizes the infected cell to inhibition of its SL partner by an inhibitory drug.

Synthetic lethality is conventionally described as a type of genetic interaction between two nonessential genes that participate in a parallel or redundant process to carry out an essential function, where mutations in either gene alone does not affect cell viability, but mutations in both genes result in cell or organismal death (Fig. 5; Nijman, 2011). This SL concept has been exploited in anti-cancer drug development and treatment (Fig. 5 B; Farmer et al., 2005; Kaelin, 2005; Mendes-Pereira et al., 2009; Turner et al., 2008; Wiltshire et al., 2010). For example, loss-of-function mutations in the DNA repair genes encoded by breast cancer type 1 and 2, *BRCA1* and *BRCA2,* cause breast and ovarian cancer but exhibit enhanced sensitivity to inhibitors of poly ADP-ribose polymerase (*PARP*), another DNA repair enzyme (Farmer et al., 2005). PARP inhibitors selectively killed cancerous cells carrying the loss-of-function *BRCA* mutation while sparing noncancerous cells (Bryant et al., 2005) and in a clinical trial, PARP anticancer drugs showed a significantly longer progression-free period in patients with breast cancer (Litton et al., 2018).

Here, we extend the idea of exploiting synthetic lethality to host–pathogen interactions specifically involving virus-induced hypomorphs (Fig. 5 C) and their SL partners. In poliovirus, the viral replication complex protein 3A physically interacts with GBF1 (Fig. 2; Belov et al., 2008; Teterina et al., 2011) and re-localizes it to poliovirus replication complexes (Richards et al., 2014), rendering it a hypomorph (Fig. 3). We show here that this infection-induced hypomorphic state sensitizes cells to disruption of the *GBF1*-SL partner, *ARF1* (Fig. 4). Thus, cells depleted of ARF1 are selectively killed when infected with the poliovirus replicon. In principle, SL partners of any number of vi-hypomorphs could be targeted with drugs in a similar manner to selectively disrupt infected host cells, shutting down the viral factory, while leaving uninfected cells relatively unscathed (Fig. 5 C).

We focused this proof-of-concept on a hypomorph generated by a viral protein interacting with a host cell protein. If a viral-host protein interaction causes a hypomorph, there are expected to be many potential SL targets. An average gene participates in ∼100 negative (SL/sick) interactions (Costanzo et al., 2016), and ideal candidates among this group would not have adverse effects when disrupted (so uninfected cells are not affected by treatment); be specifically druggable; proviral (to realize a potentially synergistic effect); and lack redundant isoforms. *ARF1* does not meet all these criteria. It has several isoforms, making it a challenging drug target and it is much more abundant than GBF1 (>100-fold; Wiśniewski et al., 2014), so functional depletion to meet a SL threshold can be difficult. Nonetheless, as GBF1 is a common target of many viruses, and it is required early in infection, it is tempting to speculate that other SL interactors of *GBF1*, might be candidates as broad-spectrum host-based antivirals.

Exploiting the SL concept is not limited to hypomorphs resulting from viral-host protein interactions. Many processes are disrupted during an infection cycle generating a new “infected cell state,” which could also be targeted (Mast et al., 2020). Ideally, the early stages of infection should be targeted, to ensure disruption of the host cell disrupts viral replication and viral spread. Indeed, many viruses target the ability of host cells to “commit suicide,” presumably ensuring the maintenance of the viral factory.

In the future, it would be possible to predict and prioritize potential SL antivirals using CRISPR screens and systems-level modeling approaches utilizing the existing and emerging databases of virus-host PPIs, quantitative proteomics, and human SL interactions. Our strategy to target SL interactions of the vi-hypomorph has potential to change the current paradigm for host-based therapeutics that can lead to broad-spectrum antivirals and can be applied to other intracellular pathogens.

## Materials and methods

### Cell culture and plasmids

HeLa cells (ATCC CCL-2) and HEK293-FT (Thermo Fisher Scientific) were cultured at 37°C in 5% CO_2_ in medium composed of high glucose Dulbecco’s modified eagle medium (DMEM; Gibco) supplemented with 10% (v/v) heat-inactivated fetal bovine serum (VWR), 1× penicillin/streptomycin (Thermo Fisher Scientific), 20 mM L-glutamine (Gibco), 1× nonessential amino acids (Gibco), 1× sodium pyruvate (Gibco), and 10 mM HEPES buffer (Gibco; complete media).

Cell line authentication was provided by the American Type Culture Collection and Thermo Fisher Scientific. In general, cells were passaged 5–10 times and periodically tested for contamination using MycoAlert Mycoplasma Detection (Lonza) kit.

A doxycycline-inducible Cas9 clonal cell line of NALM-6 cells was cultured at 37°C in 5% CO_2_ in RPMI-1640 medium supplemented with 10% (v/v) heat-inactivated fetal bovine serum, as described (Bertomeu et al., 2018).

Agarose stabs of *E. coli* (DH10B) harboring custom-made mammalian expression vector pD2109-EF1 were purchased from ATUM. pD2109-EF1 encodes poliovirus protein 3A with a modified FLAG tag (DYKDDDYK) inserted at the N-terminus. The modified FLAG tag (referred here as FLAG*), contains a tyrosine (Y) residue at position 7 instead of aspartic acid residue (D) found in a typical FLAG tag sequence (Teterina et al., 2011). pXpA-Ren-3A-FLAG-Y is a replicon encoding cDNA of poliovirus type I Mahoney under control of a T7 RNA polymerase promoter with a 939 nucleotide sequence encoding *Renilla* luciferase cloned in place of sequence encoding virus capsid, and a 24 nucleotide sequence encoding FLAG* appended to the 3′ end of 3A (Teterina et al., 2011; Viktorova et al., 2018). A complete sequence of the 3A-FLAG*protein with the inserted FLAG* tag is as follows: GPLQYKDYKDDDYKDLKIDIKTSPPPECIN​DLLQAVDSQEVRDYCEKKGWIVNITSQVQTERNINRAMTILQA​VTTFAAVAGVVYVMYKLFAGHQ.

Plasmid DNA was purified from the agarose stabs using NucleoBond Xtra Midiprep kit (Macherey Nagel) by following the manufacturer’s protocol. 3A-FLAG* protein was expressed in HeLa cells by plasmid DNA transfection. We used an empty plasmid pLKO1.puro with comparable size to pD2109-EF1 as a control for transfections.

Bacterial glycerol stocks of MISSION shRNAs were purchased from Sigma Aldrich for: *ARF1* (clone ID: TRCN0000039874, TRCN0000039875), *ARF4* (TRCN0000298174, TRCN0000047940), *MSMO1* (TRCN0000230198, TRCN0000046245), *CSK* (TRCN0000-199500, TRCN0000000804), *HSP90* (TRCN0000008747, TRCN00-00315415), and *PRKAA1* (TRCN0000000861, TRCN0000000859). shRNA plasmid DNA was purified using NucleoBond Xtra Midiprep kit by following the manufacturer’s protocol.

### Chemogenomic screening and data analysis

**Figure S1.**
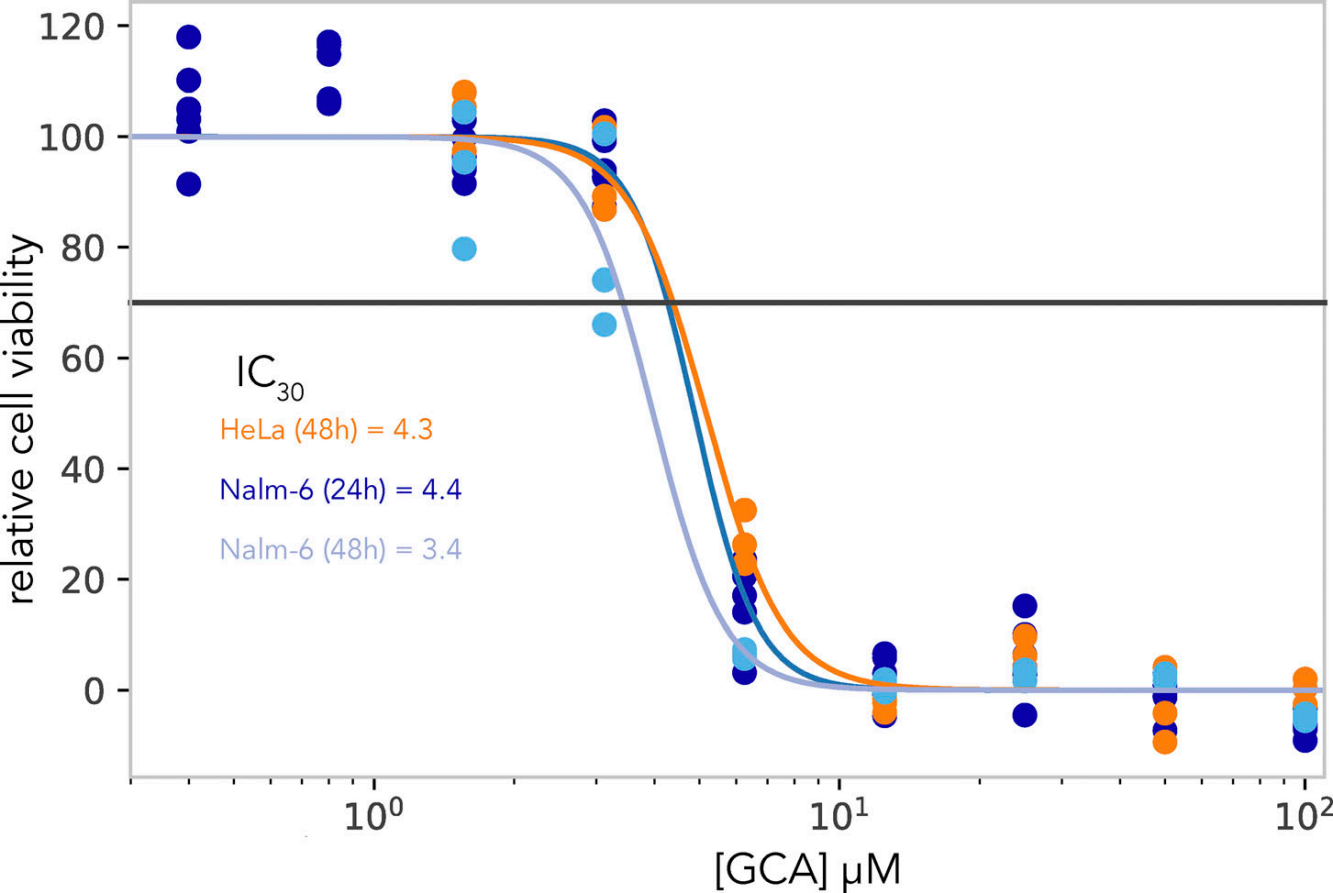
**GCA dose-response assay in Nalm6 and HeLa cells.** Nalm-6 and HeLa cells were incubated with serially diluted GCA or DMSO (4×) and cellTiterBlue reagent was added after 24 and 48 h for Nalm-6 and 48 h for HeLa cells. Metabolically active cells convert the reagent into a fluorescent product, and the fluorescence intensity recorded by a plate reader is directly proportional to the number of live cells. The fluorescence of the GCA-treated samples was normalized to the equivalent DMSO-treated controls and IC_30_ values were determined using synergy software. IC_30_ values of Nalm-6 cell line over time were averaged (IC_30_ average = ∼4.0 µM), to determine the concentration of GCA to be used in the chemogenomic drug screening assay. The IC_30_ value of HeLa cells at 48 h was comparable to that of the Nalm-6 cells.

Genome-wide custom extended-knockout (EKO) pooled library was created in a B-cell lymphoma line using a published protocol (Bertomeu et al., 2018). Briefly, a clone of the NALM-6 cells expressing Cas9 under a doxycycline-inducible promoter was transduced with the 278K sgRNAs followed by selection over Blasticidin, and induction of knockdown of genes with 2 µg/ml doxycycline over a 7-d period. At that time (day 0), the EKO library was split into separate flasks, one containing 4 µM GCA, three containing media alone and two containing 0.1% DMSO, and each library flask was grown for eight more days. 4 µM GCA was empirically determined prior to the screen to maximize both enrichment, i.e., positive selection for the rescue of compound toxicity, and depletion, i.e., negative selection for SL interactions (Fig. S1). During this period, cell counts were made every 2 d and population doublings were monitored. After each cell count, cells were diluted down to 28 million cells per flask and fresh media was added. Whereas all other samples were grown in T-75 flasks from days 0 to 8 of the screen, one of the untreated control samples was grown in a T-175 flask and was diluted down to 70 million cells every 2 d instead of 28 million. sgRNA sequences were recovered by PCR of genomic DNA, reamplified with Illumina adapters, and sequenced on an Illumina HiSeq 2000 instrument. The GCA-treated sample DNA was later re-sequenced on an Illumina Next-Seq 500 instrument to increase coverage. Illumina sequencing reads were aligned to the theoretical EKO library using Bowtie 2.2.5, with the -norc (no reverse complement) option and otherwise default parameters. sgRNA read counts were tabulated from all successfully aligned reads. Having found no significant differences between untreated and 0.1% DMSO-treated controls, we opted to add together the sgRNA read counts from all control samples to generate a more robust estimate of the expected sgRNA frequency distribution. We used RANKS (Robust Analytics and Normalization for Knockout Screens; Bertomeu et al., 2018) with default parameters to generate gene scores P value and FDR values, comparing the sgRNA read counts of the GCA-treated sample to those of the controls (Table S1). We also calculated gene-level log_2_ fold-changes in sgRNA representation by first summing across each sample the reads of all (usually 10) sgRNAs targeting the gene to calculate a single ratio normalized to the ratio of total aligned read counts per sample (Table S1). This approach effectively downweighs less well-represented guides in contrast to the traditional approach of taking the average of the individual sgRNA fold-changes. Reported gene essentiality and essentiality rank in Table S1 are from a previous screen (Bertomeu et al., 2018).

Ontology biological process enrichment analysis was performed on the putative *GBF1*-SL genes (FDR < 0.05) using ClusterProfiler (Yu et al., 2012) where the list was analyzed against the entire KO genes from the CRISPR library to functionally classify the SL genes.

**Figure S2.**
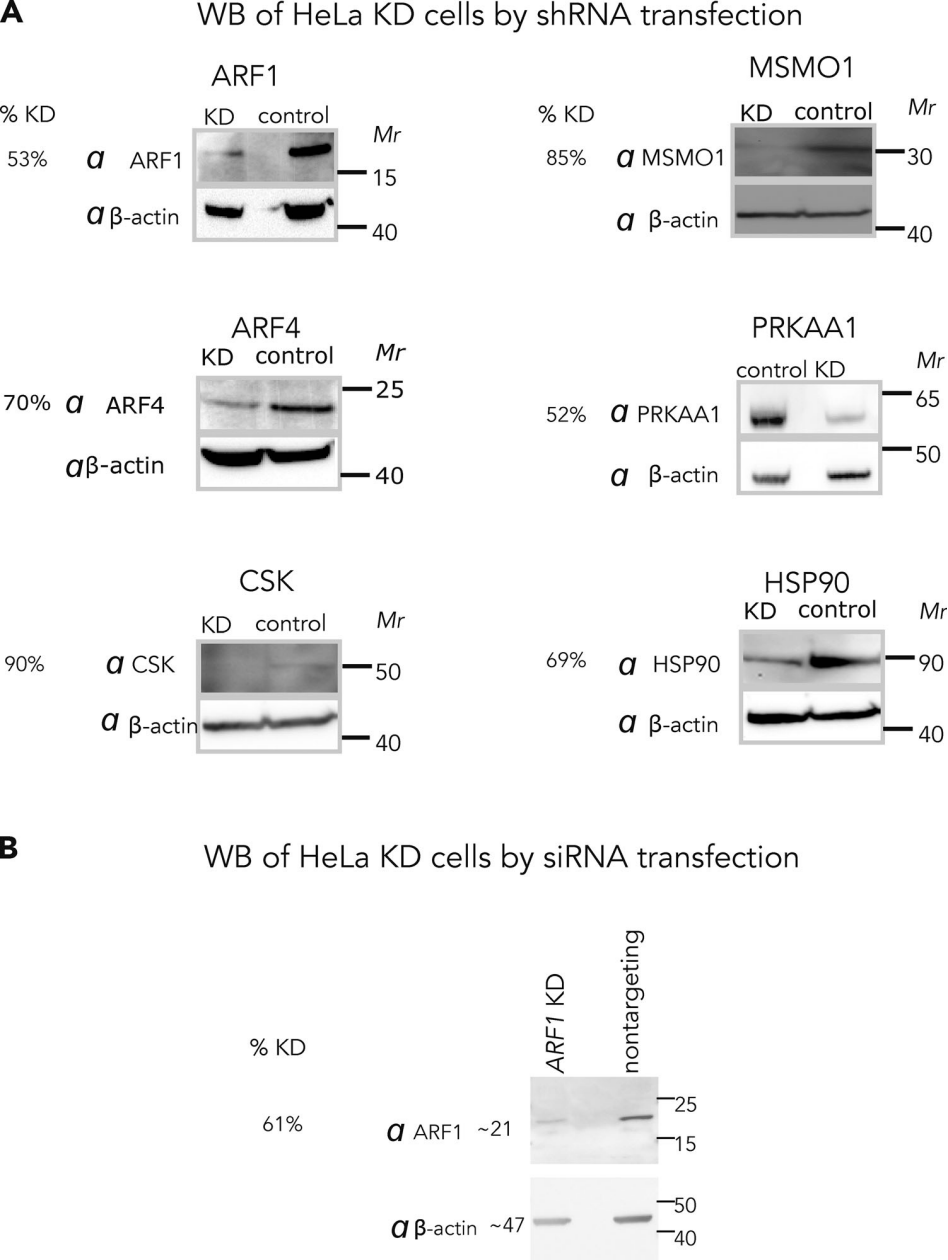
**Validation of shRNA and siRNA-mediated knockdown of genes. (A)** Four druggable putative synthetic lethal partners of GBF1: *ARF1, PRKAA1*, *CSK*, and *HSP90*; a putative GBF1 suppressor, *ARF4*; and a gene showing no GCA-induced chemogenomic interactions with *GBF1*, *MSMO1*, were silenced in HeLa cells via shRNA-mediated lentivirus transductions and selected on puromycin for 72 h. Cell pellets from each knockdown cell-line were collected, lysed, and the total protein concentrations were measured using a bicinchoninic acid assay. Equal amounts of total protein from the control and knockdown cells were resolved by SDS-PAGE, transferred to PVDF and probed with the indicated antibodies. **(B)** For experiments with siRNA, HeLa cells were transiently transfected with siRNAs targeting *ARF1* and a nontargeting control. After 48 h post-transfection, the control and KD cells were harvested and replated for IF imaging experiment (Fig. 4, D–F, main text). Cell pellets were collected and used for WB analysis as described above. β-actin was used as a loading control. Protein depletion relative to the respective loading control was calculated using ImageJ software and the resulting percent knockdown efficiencies (% KD) are reported. Source data are available for this figure: SourceData FS2.

The list of 70 genes (53 *GBF1*- SL partners and 17 *GBF1* suppressors) passing the FDR cut off (<0.05) was submitted to the STRING database (Szklarczyk et al., 2019) to map evidence-based PPIs with the active interaction sources: textmining, experiments, databases, co-expression, neighborhood, gene-fusion, and co-occurrence. The resulting PPI network was visualized using Cytoscape (3.8.2; Shannon et al., 2003) in a radial layout, with proteins denoted as circles and interactions as edges. The 70 genes were also searched against a virus-host PPI database, VirHostNet (v2.0; Navratil et al., 2009), to identify known interactors of virus proteins.

### 10× Lentivirus stock preparation

Glycerol stocks of the validated MISSION shRNA vectors for *ARF1*, *PRKAA1*, *HSP90*, *CSK*, *MSMO1* and *ARF4* were obtained from Sigma-Aldrich. 10× stocks of non-replicating lentiviral stocks were generated by transfection of HEK293-FT cells as follows: 4 × 10^6^ HEK293-FT cells were plated on poly-L-lysine-coated 10-cm dishes to achieve 70–80% confluency at time of transfection. The following day, transfection mixtures were prepared by mixing 20 µl Polyethylenimine MAX (Polysciences, Inc.) prepared at 1 mg/ml, together with 4.75 µg of transgene shRNA constructs, 1.5 µg of viral envelope plasmid (pCMV-VSV-G), and 3.75 µg of viral packaging plasmid (psPax2). After incubating for 10 min at room temperature in DMEM, transfection complexes were added dropwise to cells. After overnight incubation, cells were washed to remove the transfection mixture and replaced with 10 ml of pre-warmed media. Lentivirus-containing supernatant was harvested 48 h later, centrifuged for 5 min at 900 *g* to remove cell debris, passed through 0.45-µm syringe filters, and collected by centrifugation for 4 h at 78,900 *g*. Supernatants were decanted and pelleted lentiviruses were re-suspended in 0.1 ml Opti-MEM (Gibco) to obtain 10× lentivirus concentrates and stored at −80°C until use. A similar protocol was used to prepare 10× lentivirus stocks of 3A-FLAG*.

### shRNA-mediated gene knockdowns

To induce knockdown of the top putative GBF1 SL genes, 3 × 10^5^ HeLa cells were transduced with lentiviral supernatants in 6-well plates. At time of plating, 10× lentivirus concentrates were diluted in 1 ml of Opti-MEM containing 8 × 10^−3^ µg/ml of polybrene (Sigma-Aldrich) and incubated overnight at 37°C. The following day, the transfection mix was replaced with 2 ml of pre-warmed complete media and incubated for 24 h. To select for cells with stable integration of shRNA transgenes, overnight media was replaced with complete media containing 1.5 µg/ml puromycin. Cells were selected for at least 3 d prior to experiments. Stably silenced knockdown cell lines (*ARF1* KD, *HSP90* KD, *CSK* KD, *PRKAA1* KD, *MSMO1* KD, and *ARF4* KD) were harvested by trypsinization in 0.25% trypsin-EDTA, washed in pre-warmed PBS, cells were counted, and a small aliquot of cells was saved for Western blot analysis to verify protein level knockdown efficiencies for each gene (Fig. S2 A).

### siRNA-mediated transient knockdowns for selective killing assay

Dicer-substrate siRNAs targeting ARF1 (design id: hs.Ri.ARF1.13) and a nontargeting control (TriFECTa RNAi Kit) were ordered from Integrated DNA Technologies. 12.5 µl of the 2 µM of DsiRNAs were mixed with 7.5 µl Lipofectamine 3000 reagent (Thermo Fisher Scientific) and 250 µl of Opti-MEM. Dicer-substrate siRNA transfection complexes were allowed to form for 15 min and 2 ml of HeLa cells at (2 × 10^5^ cells/ml) were directly added to the transfection mix and plated in a 6-well plate. 48 h post-transfection, KD cells were harvested, counted, and a small aliquot of cells per KD was saved for Western blot analysis to verify protein level knockdown efficiencies (Fig. S2 B).

### Western blot analysis

Cell pellets of the KD cell lines were resuspended in a chilled IP lysis buffer (20 mM HEPES-KOH, pH 7.5, 1% [w/v] Triton X-100, 0.5% [w/v] sodium deoxycholate, 110 mM potassium acetate, 2 mM MgCl_2_, 25 mM NaCl, and 1× cOmplete protease inhibitor cocktail [Roche]) and lysed by sonicating for 1 min using a probe sonicator (QSonica) operated at an amplitude of 10 with 10 s on-off cycles. Lysates were centrifuged at ∼100,000 *g* for 5 min and supernatants were transferred into a fresh tube. Protein concentrations were determined by bicinchoninic acid assay (Thermo Fisher Scientific) and working solutions of lysates at concentrations of 15–30 µg total proteins per 30 µl were prepared with 1× lithium dodecyl sulfate (LDS) sample buffer with reducing reagent (NuPAGE, ThermoFisher Scientific) followed by heating at 70°C for 20 min on a Thermomixer (Eppendorf). 30 µl of the reduced lysate was loaded per well on 4–12% or 12%, for *ARF1* KD and *ARF4* KD, Bis-Tris gels (NuPAGE, Thermo Fisher Scientific) and protein bands were resolved at a constant voltage of 170 V for 1 h. Protein bands were transferred on PVDF membranes using Xcell2 blot module (Thermo Fisher Scientific) for 2 h at a constant voltage of 37 V and membranes were blocked in 5% (w/v) milk in TBST for 1 h at room temperature. After blocking, membranes were incubated with primary antibodies (Abcam and GeneTex) against proteins of interests: ARF1 (ab58578 at 1:1,000 dilution), ARF4 (ab190000 at 1:1,000 dilution), HSP90 (GTX101448 at 1:1,000 dilution), CSK (GTX107916 at 1:500 dilution), CSNK2A (13–453 at 1:500 dilution), PRKAA1 (ab32047 at 1:1,000 dilution), and MSMO1 (ab116650 at 1:500 dilution) and washed thrice in TBST buffer before incubating with appropriate HRP-conjugated secondary antibodies (goat α-mouse or α-rabbit; 1:2,500 dilution). Following incubation, membranes were washed and developed using chemiluminescent substrates (Advansta WesterBright). Images were acquired using a FluorChem imager (Protein Simple), and membranes were stripped using a stripping buffer (Thermo Fisher Scientific), blocked, incubated with HRP-α-β-actin (ab49900; 1:25,000) and imaged as before. Relative protein depletion (% knockdown efficiency) was quantitated by dividing the protein band intensity with the protein band intensity of the actin control, using ImageJ software. Images were cropped, adjusted for brightness and contrast, and labeled using Adobe Photoshop and InDesign.

### FLAG*-tagged 3A lentivirus direct plasmid transfection

 FLAG*-tagged 3A plasmid DNA was directly transfected into HeLa cells for performing immunofluorescent imaging and co-immunoaffinity purification assays using TransIT transfection reagent (Mirus) by following the manufacturer’s recommended protocol. Briefly, transfection mix was prepared in a serum free Opti-MEM media by adding 3A-FLAG* DNA and TransIT reagent in a ratio of 1:3 (wt/v) and the mixture was incubated for 30 min at room temperature. After incubation, the transfection mix volume equivalent to 1 and 15 µg total DNA was added to 6 × 10^5^ HeLa cells for immunofluorescence imaging and 3 × 10^6^ cells for co-immunoaffinity purification assays, respectively.

### Flow cytometry

HeLa cells transduced with FLAG*-tagged 3A plasmid were trypsinized post 24 h using 0.05% (w/v) trypsin-EDTA and transferred into a U-bottom 96-well plate. Cells were washed twice in PBS supplemented with 10% (v/v) FBS and incubated with Live/Dead Fixable stain (excitation/emission: 416/451; Thermo Fisher Scientific) for 15 min on ice. Excess stain was removed by washing twice and cells were fixed using 4% (w/v) formaldehyde (Sigma-Aldrich) for 30 min, washed and incubated in PBS with 0.1% (w/v) Triton X-100 for 15 min to permeabilize the cells. After cell permeabilization, the cells were blocked for 1 h in PBS containing 2% (w/v) BSA and 1% (w/v) Triton X-100. Cells were then stained with R-phycoerythrin (PE)-conjugated α-FLAG (637310, 1:1,000 dilution; Biolegends) for 1 h on ice. After staining, cells were washed thrice in the blocking buffer and analyzed on a LSRII flow cytometer (BD Biosciences). The percentage of cells expressing 3A-FLAG* were determined by analyzing the flow cytometry data using FlowJo software (Tree Star, Inc.). Cell populations were filtered using the forward and side scatter to remove cell debris and cell doublets. The remaining single cell subpopulation was then divided using the intracellular PE staining into 3A-FLAG* positive and negative populations and percentages of positive and negative cells of the total single cells were reported. Experiments were performed in triplicate.

### Assessment of GBF1 vi-hypomorph formation by immunofluorescence microscopy

HeLa cells were plated in 12-well plates containing 12-mm no.1.5 circular glass coverslips (Fisherbrand) at a cell density of 6 × 10^4^ per well and two wells were transfected with FLAG*-3A or an empty plasmid control. Cells were fixed 24-h post-transfection with 2% (w/v) formaldehyde (Sigma-Aldrich) for 30 min, permeabilized with 0.1% (w/v) Triton X-100, and blocked with 2% (w/v) BSA, 0.1% (w/v) Triton X-100 in PBS (blocking buffer). After blocking, cells were incubated with rabbit α-GBF1 (ab86071, 1:1,000 dilution; Abcam) and mouse α-FLAG (F1804; 1:200 dilution; Sigma-Aldrich) for 1 h followed by 1 h staining with secondary antibodies goat α-rabbit AlexaFluor-488, and goat α-mouse AlexaFluor-594 (Invitrogen), used at a 1/1,000 dilution. The coverslips were mounted on Superfrost microscope slides (Fisherbrand), nuclei were stained with DAPI, and the cells were cleared with Prolong Glass (Invitrogen). Images were acquired with a 100× NA 1.4 objective (Olympus) on a DeltaVision Elite High-Resolution Microscope (GE Healthcare Life Sciences). Fluorescence excitation was driven by an Insight SSI solid state light engine (Cytiva), and fluorescence emission was collected by a CoolSnap HQ2 CCD camera (Photometrics). The sides of each CCD pixel are 6.45 µm. Image *z* stacks were acquired with 0.2 µm steps and 25–27 images per stack. Images were deconvolved with a classic maximum likelihood estimation algorithm using Huygens software (Scientific Volume Imaging) and experimentally determined point spread functions captured by imaging PS-Speck beads (Invitrogen) under experimental conditions, as done previously (Mast et al., 2018; Vijayan et al., 2019).

### Quantification of GBF1 vi-hypomorph formation

Images were processed using Imaris software (Bitplane) to quantify the number of GBF1 puncta per cell. Initial cell segmentation was performed by summing the fluorescence intensities from all channels and using the “Surface” command to threshold the images. This segmentation was refined using the “Cell” command. Cell nuclei were defined using the DAPI channel and cell boundaries defined using a watershed algorithm seeded by the “one nucleus per cell” function to split touching cells. Next, the GBF1-488 channel was selected for detecting GBF1 puncta using the “detect vesicle” function. Statistical values for “vesicle intensity sum” and “vesicle distance to closest nucleus” for each GBF1 puncta per cell were exported for 42 cells from control samples, and from 13 cells of 3A-FLAG* transfected cells. Distances of GBF1 puncta from the nearest nucleus in 3A-FLAG* cells were compared to distances from control cells using Wilcoxon rank-sum tests. Comparisons were made using all observed puncta and, separately, using only puncta more than 20 mm from the closest nucleus.

### Affinity purification

HeLa cells were transfected with 1 µg DNA encoding FLAG*-3A or an empty control plasmid. At 24 h post-transfection, cells were lysed using mild sonication in IP lysis buffer (20 mM HEPES, 1% [w/v] Triton X-100, 2 mM magnesium chloride, 25 mM sodium chloride, 110 mM potassium acetate, and 0.2% [v/v] antifoam B), and clarified by centrifugation at 100,000 *g* for 3 min. Total protein concentrations were measured using a BCA assay (Pierce) and 100 µg of lysate from each sample was used in the affinty purification. 8 µg of α-FLAG (F3165; Sigma Aldrich) were conjugated to 10 mg epoxy-coated M-270 magnetic beads (Thermo Fisher Scientific; Cristea and Chait, 2011). The α-FLAG-conjugated beads were washed and re-suspended in IP lysis buffer, and 3-mg bead aliquots were added to the clarified lysates. Lysates were incubated with magnetic beads overnight at 4°C. After three washes with IP lysis buffer, bound proteins were eluted with 50 μl 1 × LDS sample buffer. 90% of the eluate was loaded on a 4–20% Bis-Tris NuPAGE gel and the remaining 10% loaded on a 3–8% Tris-Tricine gel to resolve GBF1 and 3A-FLAG, respectively. 1% of the input lysate and last wash was also run on each gel. Proteins were transferred to PVDF membranes and immunoblotted with α-GBF1 (ab86071, at 1:1,000 dilution; Abcam) and HRP conjugated α-FLAG (A8592; 1:2,000 dilution; Millipore Sigma). The experiment was performed in triplicate.

### Cell viability assay

Cell viability assays were performed in 96-well plates using the CellTiter Blue reagent (Promega). Golgicide A was purchased from Cayman Chemicals (product # 18430). Working concentrations of GCA were prepared by diluting a 10 mM DMSO-solubilized stock in complete media. Equimolar solutions lacking the drug were prepared by diluting neat DMSO. Cells were incubated in 100 µl of GCA or DMSO alone working solutions for 48 h before 20 µl of the CellTiter Blue reagent was added, and fluorescence was measured 4-h post-addition using a Synergy HTX Multi-mode plate reader (BioTek). The metabolically active cells convert the blue redox reagent into its fluorescent product with the number of live cells directly proportional to the intensity of the fluorescent product. Fluorescence measurements from the drug-treated samples were normalized using the signal from matched DMSO alone-treated samples to normalize for any cell-line specific effects of the DMSO solvent on cell viability.

For the viability assay validating *GBF1*-SL candidates, 2 × 10^4^ cells of each gene KD cell line (*ARF1* KD, *HSP90AB* KD, *CSK* KD, *PRKAA1* KD, *ARF4* KD, *MSMO1* KD) were seeded per well in 200 µl media. The next day, the media was replaced with 100 µl of GCA or DMSO at 1.5 and 4 µM, incubated for 48 h, and cell viability was measured as described above. Normalized cell viabilities of the KD cells were compared to that of the *MSMO1* KD controls.

For generating GCA dose response curves we used an automated high throughput liquid handling system (PipetteMax, Gilson) for co-plating cells with the drug. Stock solutions of GCA or DMSO were prepared in complete media at a concentration of 200 µM, and serially diluted in cell-containing media of *ARF1* KD and *MSMO1* KD (4 × 10^5^ cells/ml) to obtain 0–100 µM of GCA/DMSO with 2 × 10^4^ cells/well, plated in triplicate. Cell viabilities were measured 48 h post-incubation as described above. GCA dose–response curves were plotted with synergy (Mast et al., 2021; Wooten and Albert, 2021).

For the proof-of-concept SL viability experiments, 6 × 10^4^ cells of *ARF1* KD and *MSMO1* KD were seeded in a 6-well plate and transfected the following day with 3A-FLAG* or an empty plasmid control. After 24 h post-transfection, cells were harvested, counted, and plated in 96-well plate at a density of 5,000 cells per well. The remaining cells were processed for flow cytometry analysis to measure transfection efficiencies. Cell viabilities were measured 48 h post-transfection using CellTiter Blue reagent, as described above. Absolute cell viabilities of *ARF1* KD and *MSMO1* KD cells were compared using a multiple *T* test, i.e., the two-stage step-up method of Benjamini et al. (2006), in GraphPad Prism 8. The experiment was performed in triplicate.

### Selective killing assay

Selective killing assays were performed using immunofluorescence microscopy. *ARF1* KD and nontargeting control HeLa cells were seeded at a 30,000 cells/0.1 ml in three poly-*L*-lysine-coated #1.5-glass-bottomed 96-well plates (Corning) using an automated high-throughput liquid handling system for uniform cell plating. Cells were transfected with 25 ng of poliovirus replicon using Lipofectamine RNAiMAX (Thermo Fisher Scientific), following the manufacturer’s protocol. Briefly, 25 ng of poliovirus replicon mRNA and the transfection reagents were mixed in Opti-MEM and incubated for 5 min, and the complex mixture was directly added per well and plates were incubated for 8, 16, and 24 h. Time-matched cells transfected with RNAiMAx alone were used as controls. At each time point, media containing the replicon was removed and cells were washed with PBS and fixed in 0.1 ml 4% (w/v) formaldehyde (Sigma-Aldrich) for 10 min at room temperature. Fixed cells were washed with PBS and permeabilized using 0.5% (v/v) Triton X-100 for 10 min at room temperature, following 1 h blocking on ice in 0.5% (v/v) Triton X-100 with 2% (w/v) BSA in PBS (blocking buffer). After blocking, cells were incubated with mouse α-FLAG (F1804; 1:200 dilution; Sigma-Aldrich) overnight followed by 1 h incubation with secondary goat α-mouse AlexaFluor-594 (Invitrogen; 1:500 dilution), Phalloidin-iFluor 488 (ab176753; 1:2,000; Abcam). After staining, cells were washed in blocking buffer, mounted in an aqueous mounting media with DAPI (ab104139; Abcam), and plates were stored at 4°C until analysis. Images were acquired with a 20× NA 0.75 objective (Keyence) on a BZ-X800 widefield fluorescence microscope (Keyence). Fluorescence excitation was driven by a 40-W LED light source and fluorescence emission was collected by a Peltier cooled CCD camera (Keyence). The sides of each CCD pixel are 7.55 µm. DAPI fluorescence was excited with a 360/40-nm excitation filter and collected with a 460/50-nm emission filter with a 400-nm dichroic mirror. AlexaFluor-488 fluorescence was excited with a 470/40-nm excitation filter and collected with a 525/50-nm emission filter with a 494-nm dichroic mirror. AlexaFluor-594 fluorescence was excited with a 560/40 nm excitation filter and collected with a 630/75-nm emission filter with a 585-nm dichroic mirror. Image tiles (7 × 9) were acquired with 30% overlap between tiles and stitched together using Keyence software.

### Quantification of selective killing

Images were processed using Imaris software (Bitplane) to quantify the number of cells per stitched image. Cell nuclei were defined using the DAPI channel and cell boundaries defined using a watershed algorithm seed by the “one nucleus per cell” function to split touching cells.

In each instance where a parametric statistical test was used, data distribution was assumed to be normal but this was not formally tested.

### Online supplemental material

Fig. S1 contains the results from a GCA dose-response assay used to determine the concentration of GCA in the CRISPR screen reported in Fig. 2. Fig. S2 contains supporting immunoblots that show the efficiency of the shRNA-mediated protein depletion for the *ARF1*, *MSMO1*, *ARF4*, *PRKAA1*, *CSK*, and *HSP90* KD cell lines. Table S1 contains supporting data reporting the GCA CRISPR screen results for the 19,029 Refseq genes. For each gene, we report the RANKS (Robust Analytics and Normalization for Knockout Screens) score, associated P values, the FDR, the number sgRNA considered for the analysis, and the gene-level log_2_ fold changes.

## Supplementary Material

Table S1 contains supporting data reporting the GCA CRISPR screen results for the 19,029 Refseq genes. For each gene, we report the RANKS (Robust Analytics and Normalization for Knockout Screens) score, associated P values, the FDR, the number sgRNA considered for the analysis, and the gene-level log_2_ fold changes

SourceData F3 is the source file for Fig. 3.

SourceData FS2 is the source file for Fig. S2.
