# Supplementary figures and images for: Viral protein engagement of GBF1 induces host cell vulnerability through synthetic lethality
Source: J Cell Biol. 2022 Oct 28;221(11):e202011050. doi: 10.1083/jcb.202011050 (PMC9623979; doi:10.1083/jcb.202011050)

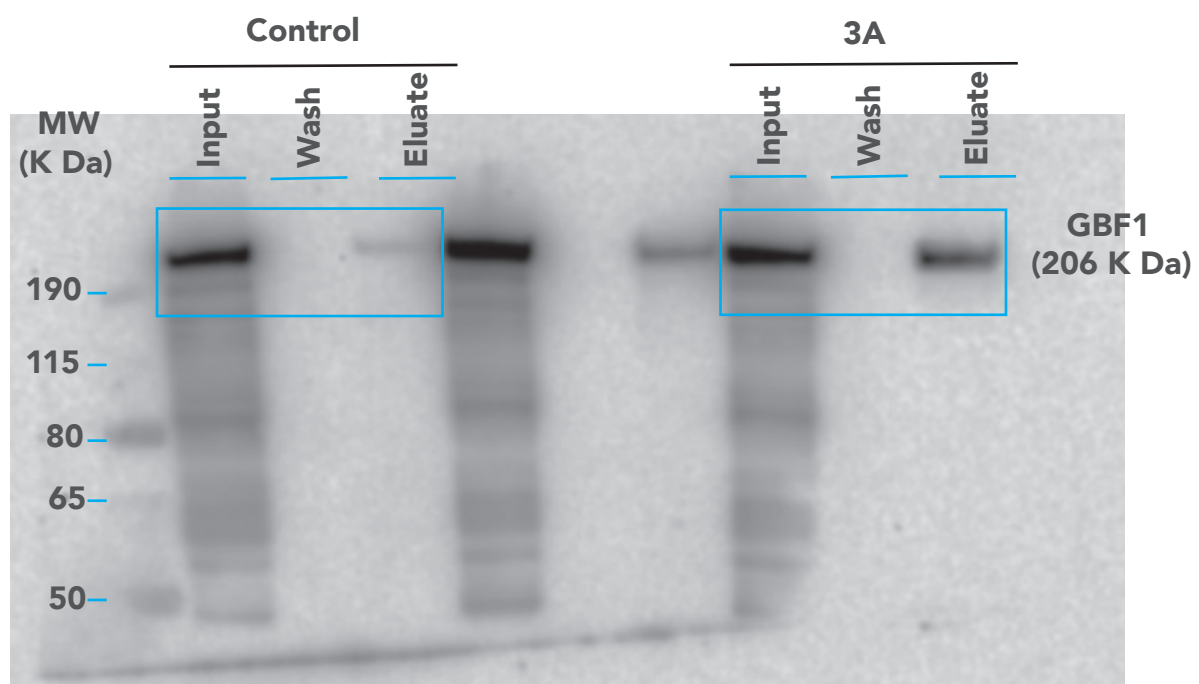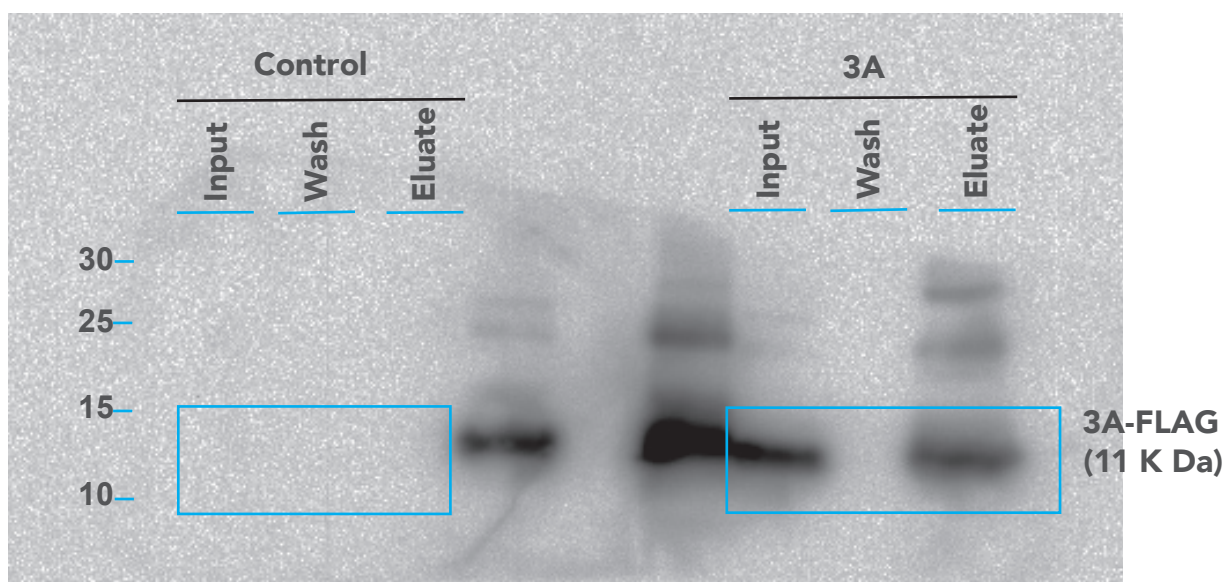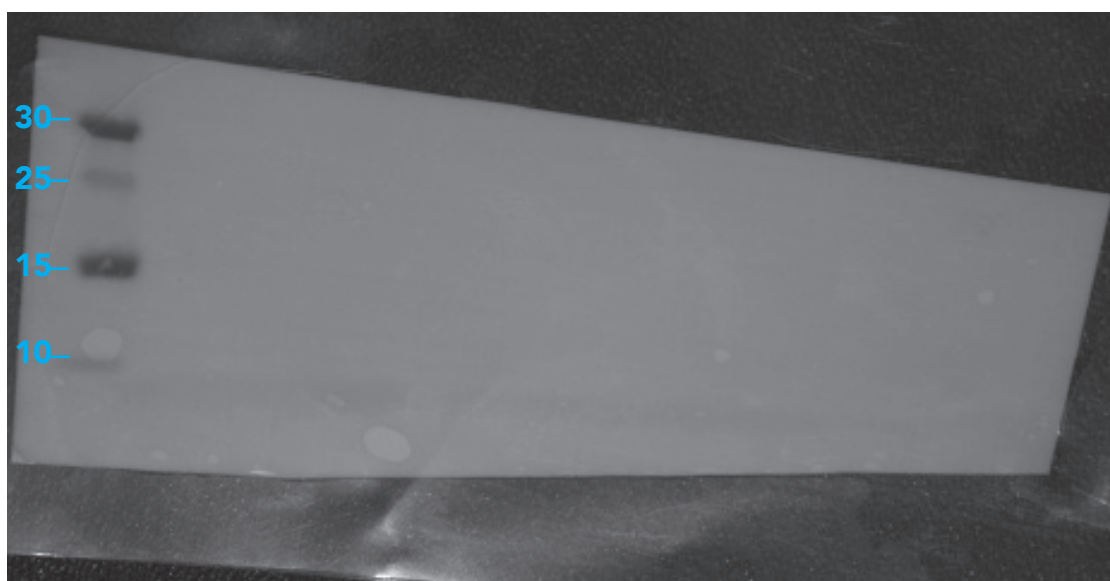

Supplement: SourceData F3 — is the source file for Fig. 3. [file JCB_202011050_SourceDataF3.pdf]

**A**

## shRNA-mediated KD

**WB : ARF1**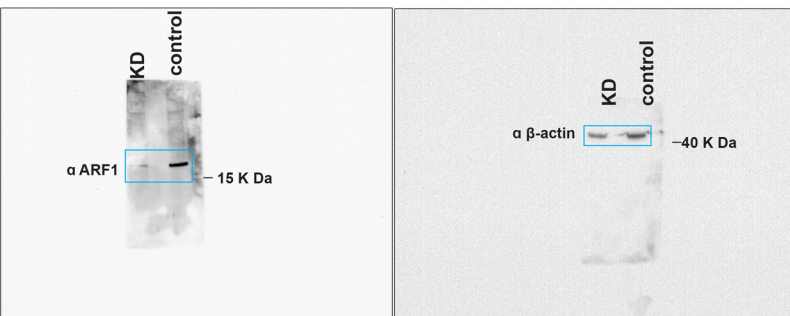**WB : MSMO1**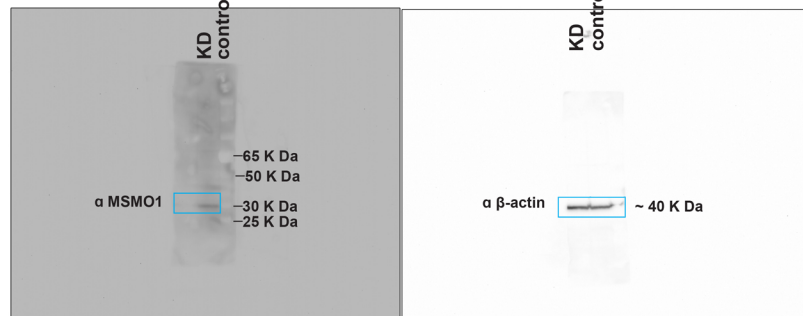**WB : ARF4**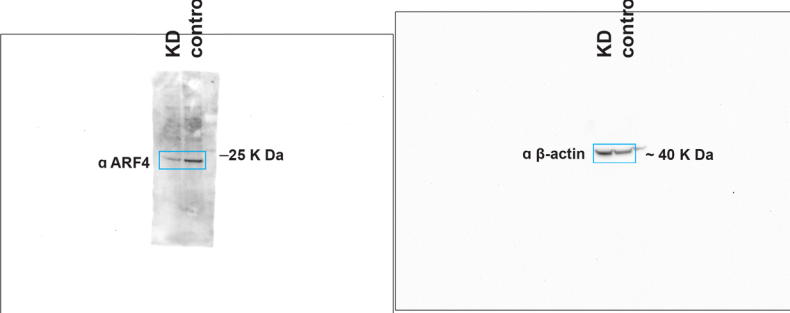**WB : PRKAA1**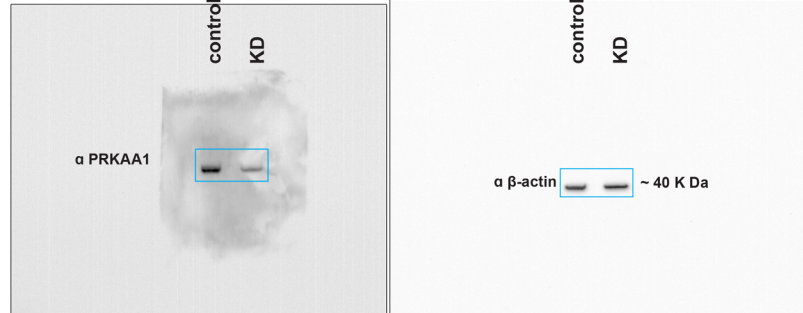**WB : CSK**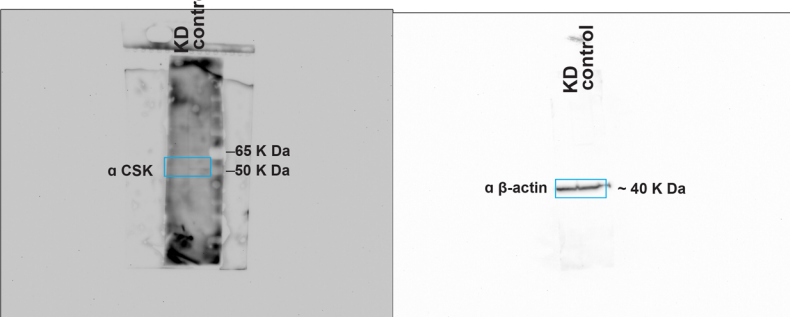**WB : HSP90**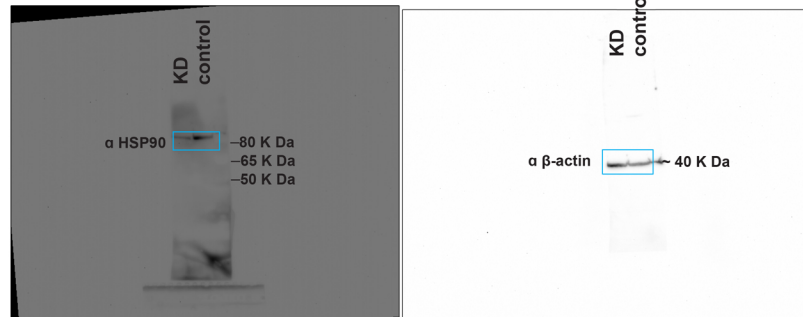

**B** siRNA-mediated KD

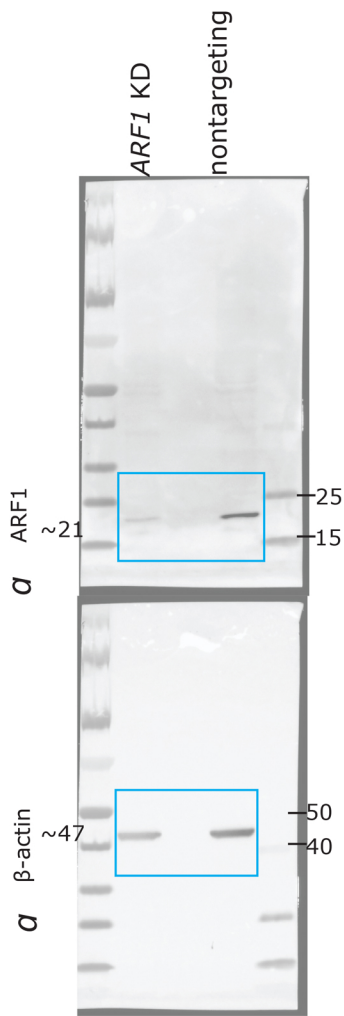

Supplement: SourceData FS2 — is the source file for Fig. S2. [file JCB_202011050_SourceDataFS2.pdf]
